# Disease-Modifying Therapies for Multiple System Atrophy: Where Are We in 2022?

**DOI:** 10.3233/JPD-223183

**Published:** 2022-07-08

**Authors:** Victoria Sidoroff, Pam Bower, Nadia Stefanova, Alessandra Fanciulli, Iva Stankovic, Werner Poewe, Klaus Seppi, Gregor K. Wenning, Florian Krismer

**Affiliations:** a Department of Neurology, Medical University of Innsbruck, Innsbruck, Austria; b The Multiple System Atrophy Coalition, Inc., McLean, VA, USA; c Neurology Clinic, University Clinical Center of Serbia, School of Medicine, University of Belgrade, Belgrade, Serbia

**Keywords:** Atypical Parkinson’s disease, clinical trials, disease modification, multiple system atrophy

## Abstract

Multiple system atrophy is a rapidly progressive and fatal neurodegenerative disorder. While numerous preclinical studies suggested efficacy of potentially disease modifying agents, none of those were proven to be effective in large-scale clinical trials. Three major strategies are currently pursued in preclinical and clinical studies attempting to slow down disease progression. These target α-synuclein, neuroinflammation, and restoration of neurotrophic support. This review provides a comprehensive overview on ongoing preclinical and clinical developments of disease modifying therapies. Furthermore, we will focus on potential shortcomings of previous studies that can be avoided to improve data quality in future studies of this rare disease.

## INTRODUCTION

Multiple system atrophy (MSA) is a fatal, adult-onset and rapidly progressive neurodegenerative disease characterized by autonomic failure, ataxia, and parkinsonism in any combination [1, 2]. Two motor phenotypes are recognized in MSA: A parkinsonian variant (MSA-P) featuring a poorly levodopa-responsive akinetic-rigid syndrome and a cerebellar variant (MSA-C) presenting with broad based gait, limb ataxia, scanning dysarthria, and cerebellar oculomotor dysfunction. Apart from these motor features, MSA is associated with autonomic failure (urogenital, cardiovascular or both) as well as several other motor and non-motor features including dystonia, pyramidal signs, REM-sleep behavior disorder or stridor [3].

Neuropathologically, misfolded α-synuclein forms insoluble aggregates, termed glial cytoplasmatic inclusions (GCIs), with consecutive microglial activation and release of pro-inflammatory cytokines and oxygen reactive species [4, 5]. Although α-synuclein is primarily an intracellular protein, a variety of α-synuclein species can be found in cerebrospinal fluid (CSF) of patients with α-synucleinopathies [6]. Preclinical studies in mice which developed α-synuclein inclusion pathology after being inoculated with brain homogenates from MSA patients, suggest that abnormally folded α-synuclein may drive the spread of MSA-related pathology from cell-to-cell in a prion-like fashion [7].

**Fig. 1:**
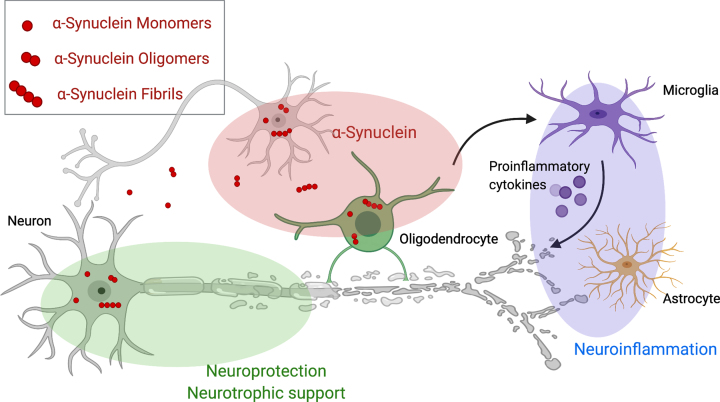
Therapeutic targets for disease modifying therapies in multiple system atrophy. This figure demonstrates pathological mechanisms underlying Multiple system atrophy and potential disease modifying targets including aggregation, spreading and clearance of α-synuclein (red), neurotrophic support (green) and the cascade of neuroinflammation (violet). Created with BioRender.com.

Thus far, potential disease-modifying therapies (DMT) have failed in clinical trials, but numerous DMTs are currently in clinical development for MSA and target different key abnormalities of the neurodegenerative cascade in MSA (as illustrated in Fig. 1). Alpha-synuclein is the most obvious therapeutic target and treatment strategies focus on the aggregation, the spreading and the clearance of (misfolded) α-synuclein. Other strategies target neuroinflammation, neurotrophic support, mitochondrial dysfunction, and excitotoxicity. In the present paper, we will review ongoing developments of DMTs in the field. We performed a non-systematic literature review using PubMed and the search terms “MSA”, “multiple system atrophy”, “treatment”, “therapy”, “disease modification”. We selected publications reporting results of disease-modifying trials that involved MSA patients and critical assessed and reviewed these reports. Studies on symptomatic therapies were excluded.

### Targeting α-synuclein

GCIs are the hallmark neuropathological finding in MSA and possibly a major contributor to the neurodegenerative cascade in MSA. In contrast to Parkinson’s disease (PD) and Lewy body dementia (DLB), where aggregated α-synuclein predominantly accumulates within astrocytes and neurons, in MSA, it mainly accumulates within oligodendroglia and to a lesser extent in neurons [8, 9]. The pathogenic cascade leading to α-synuclein aggregation and neurodegeneration of this oligodendroglioneuronal proteinopathy are poorly understood [8, 9]. Recent studies suggest an early translocation of α-synuclein to the cell nucleus [10]. Further, myelin-associated oligodendrocyte basic protein and huntingtin interacting protein 1 appear to interact with α-synuclein thriving pathogenic cascade of MSA [11]. Converging evidence suggests a prion-like spreading of misfolded α-synuclein strains as a key pathogenic event [12–21] and some authors even suggested that MSA is a prion disease [7, 22–24]. However, the latter remains a matter of intense debate [25–30].

### Clinical development

**Fig. 2:**
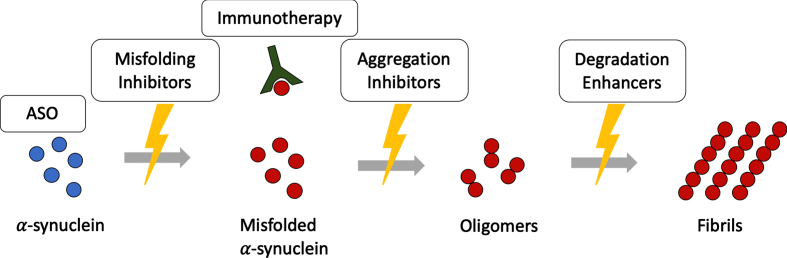
Disease modifying therapies targeting α-synuclein assemblies at different stages. Disease modifying therapies target different levels along the α-synuclein aggregation cascade. ASO, antisense oligonucleotides.

Therapies targeting α-synuclein in MSA are illustrated in Fig. 2. Table 1 gives an overview on therapeutic strategies targeting α-synuclein.

**Table 1:** Therapeutic strategies targeting α-synuclein in MSA

Mode of action	Substance	Phase	Design	Primary outcome	Results	Comments
Immunotherapy	PD01A/PD03A	Phase I	RCT	Safety & tolerability	Safe & well-tolerated	PD01A: significant immunoresponse against α-synuclein
	Lu AF82422	Phase I	RCT	Safety & tolerability	Safe & well-tolerated	In healthy controls and PD patients
Antisense oligonucleotides	BIIB101	Phase I	RCT	Safety & tolerability	–	Ongoing in MSA patients
Aggregation inhibitors	EGCG	Phase III	RCT	UMSARS part II score	Negative	Exploratory analyses suggested reduced striatal volume loss
	NPT200-11A	Phase I	RCT	Safety & tolerability	Results pending	In healthy volunteers
	Anle138b	Phase Ib	RCT	Safety & tolerability	–	Ongoing in mild to moderate PD
	ATH-434	Phase I	RCT	Safety & tolerability	Safe & well-tolerated	In healthy volunteers
	CLR01	Preclinical				Molecular tweezer
	NPT088	Preclinical				Fusion protein
	Synuclein-D	Preclinical				Small molecule
	IkT-148009	Preclinical				Small molecule
	Kallikrein-6	Preclinical				Neurosin
Degradation enhancers	Rifampicin	Phase III	RCT	UMSARS part I score	Early termination	Futility criteria were met
	Rapamycin	Phase II	RCT	UMSARS total score	Early termination	Futility criteria were met
	Lithium	Phase II	RCT	Number of SAE & nSAE	Early termination	Severe adverse events

RCT, randomized-controlled trial; UMSARS, United Multiple System Atrophy Rating Scale; SAE, serious adverse event; nSAE, non-serious adverse event; PD, Parkinson’s disease; MSA, multiple system atrophy.

### Active and passive immunization

Although there is no definite evidence that α-synuclein is absolutely essential and the only relevant pathogen for the development of neurodegeneration or clinical parkinsonism [31], the potential contribution of α-synuclein is evidenced by above mentioned preclinical studies and clinical observations that SNCA multiplications cause familiar PD [32]. Furthermore, α-synuclein induced disturbances in cellular pathways (autophagy and lysosomal pathways) [33] and the cell-to-cell transmission likely occurring through secretion via exosome release and reuptake through endocytosis provides a clear rationale for α-synuclein directed therapies warrant further (pre)clinical evaluation [34]. However, it has to be acknowledged that the majority of immunotherapeutic constructs aim to mitigate extracellular pathology and intracellular proteinopathy cannot be tackled directly through these immunotherapeutic attempts. The latter would require the development of intrabodies interfering with intracellular α-synuclein species [35].

Active immunization was used in MBP-α-synuclein transgenic mice, a model of MSA that expresses α-synuclein in oligodendrocytes, suggesting amelioration of the neurodegenerative pathology in a preclinical model of MSA. In this study, a vaccine-induced production of specific anti-α-synuclein antibodies crossing the blood-brain barrier was observed. Neurodegeneration as well as demyelination in neocortex, striatum, and corpus callosum were markedly reduced in *in vivo* models of synucleinopathies [36]. A subsequent phase I study in humans reported that AFFITOPE PD01A elicited an antibody response specific to the C-terminal region of α-synuclein and was safe and well-tolerated in patients with early PD [37]. A recent phase I trial in MSA patients revealed that both PD01A and PD03A were able to induce a significant and sustained immune response against α-synuclein with a higher responder rate in the PD01A group [38]. Future studies are required to determine whether PD01A or PD03A-induced antibodies are in fact able to modify the natural course of MSA.

Passive immunization is another promising immunotherapeutic approach. Several novel monoclonal antibodies (mAb) binding to α-synuclein with high affinity are in the therapeutic pipeline.

Lu AF82422 is a mAb proven as safe and well tolerated in a phase I trial in healthy subjects and patients with PD (NCT03611569). A phase II study investigating the safety and efficacy in patients with MSA is planned for the end of 2021 [39].

Other clinical trials investigating passive immunotherapies are currently not being investigated in MSA, but are promising against α-synuclein in PD, and therefore may also be beneficial for MSA treatment. Prasinezumab (also known as PRX002) is a mAb therapy under active development in PD. A phase I study reported that the antibody was safe and well tolerated [40]. A larger, phase II trial failed to slow motor symptom worsening in patients with PD, but showed signals of efficacy on secondary and exploratory outcomes including MDS-UPDRS subscores, MoCA, and striatal dopamine receptor binding (NCT03100149 and NCT04777331) [41].

Another α-synuclein-directed mAb is MEDI1341. This antibody was shown to block cell-to-cell transmission of preformed α-synuclein fibrils *in vitro* and was able to sequester extracellular α-synuclein *in vivo*. A phase I study to assess safety and tolerability of single ascending doses of MEDI1341 in healthy volunteers was recently completed, but the results are still pending (NCT03272165). The second phase I trial, assessing multiple ascending doses in patients with PD is ongoing (NCT04449484).

The mAb BIIB054 proved effective in reducing the α-synuclein load and improving behavioural deficits in animal models of PD and DLB [42–44]. A randomized phase I trial in healthy adults and PD patients showed good safety, tolerability, and favourable pharmacokinetic profiles [45]. However, a randomized controlled phase II study in PDs failed to meet its primary outcome and the drug development was discontinued (NCT03318523).

Altogether, active and passive immunotherapy may play a role in disease modification in MSA, but further, large-scale studies are required to confirm the neuroprotective efficacy of immunization strategies. Limitations of accessibility of intraneuronal α-synuclein aggregates not being targeted with immunization need to be overcome. Furthermore, previous immunization studies in Alzheimer’s disease have clearly demonstrated that immunisation in neurodegenerative disease may cut both ways with post-vaccination meningoencephalitis possibly occurring in a substantial number of treated patients with detrimental consequences [46]. Notably, in this trial immunisation resulted in a clearance of amyloid plaques and long-term follow-up demonstrated that patients with Alzheimer’s disease actively immunized against amyloid-β remained virtually amyloid plaque-free for 14 years [47]. However, most patients in this trial had progressed to severe dementia nonetheless [48]. Finally, it remains to be studied whether neuroinflammation contributes to neurodegeneration or protects neurons from toxic alpha-synuclein species in MSA.

### Antisense oligonucleotides

Keeping in mind that intracellular aggregation of α-synuclein play a central role in the MSA pathology, the reduction of α-synuclein production itself provides rationale for disease modification. Antisense oligonucleotide (ASO) therapy can inhibit intracellular production of α-synuclein by targeting the pre-mRNA of the SNCA gene [49]. Animal models demonstrated neuroprotective effects and a marked reduction of α-synuclein in CSF and brain tissue [49, 50]. However, there are concerns rendered by preclinical studies suggesting that a complete α-synuclein knock-out may exacerbate neuroinflammation and have detrimental effects [51–53]. Therefore, selecting the appropriate degree of treatment-induced interference with α-synuclein translation is critical.

A phase I randomized controlled trial with intrathecal application of the ASO BIIB101 in MSA patients is ongoing (NCT04165486). Another clinical trial of ASO targeting leucine-rich repeat kinase 2 (LRRK2) is currently underway (NCT03976349).

### Inhibition of α-synuclein misfolding

The small molecule Epigallocatechin gallate (EGCG), a green tea extract, binds to unfolded α-synuclein polypeptide chains and inhibits β-sheet formation, thus, preventing aggregation and prion-like spreading [54–56]. Previous studies suggested EGCG as an iron chelator conferring protection against neurotoxicity [57]. Although preclinical models showed evidence of effectiveness [58, 59], a multi-center randomized controlled phase III trial in patients with MSA failed to demonstrate disease modifying effects after 52 weeks of treatment and hepatotoxicity was observed in some patients [60]. However, an exploratory analyses of the MRI sub-study showed lower annual volume loss in striatum and precentral gyrus in EGCG treated MSA patients [61].

Another small molecule for potential MSA therapy is the α-synuclein misfolding inhibitor NPT200-11A. Preclinical studies in mouse models of PD showed beneficial effects on reducing α-synuclein pathology in the cortex and astrogliosis. Normalized striatal dopamine transporter levels and improvement of motor function were observed [62, 63]. A phase I trial in healthy subjects to determine the safety, tolerability and blood levels of NPT200-11A is completed without published results so far (NCT02606682).

### Inhibition of α-synuclein aggregation

Anle138b is a small molecule targeting intracellular oligomers of α-synuclein. Preclinical studies reported high oral bioavailability and blood–brain barrier penetration. Anle138b blocks oligomer formation without affecting the monomers of α-synuclein, therefore preserving its physiological function [64]. Behavioral improvements correlating with a 30% reduction of α-synuclein accumulation in substantia nigra *pars compacta* (SNpc) and striatum as well as a significant reduction of microglial activation were observed in a MSA mouse model [65, 66]. A phase I study in healthy volunteers to determine safety, tolerability, and blood levels of orally administered anle138b has been completed successfully and the results are pending [67]. A Phase 1b study for anle138b in patients with mild to moderate PD is currently recruiting patients (NCT04685265).

Dysregulation of iron metabolism in the SNpc promotes the aggregation of α-synuclein and production of cellular reactive oxygen species causing neuronal death [68]. First experiments with novel quinazolinone inhibitor ATH434 (previously known as PBT434) revealed reduced levels of α-synuclein and markers of oxidative stress accompanied by motor improvement in PD animal models [69]. Similar results were reproduced in transgenic MSA mice [70, 71]. In a phase I study with healthy volunteers, ATH434 was safe and well tolerated (U1111-1211-0052) and achieved CSF concentrations comparable with those associated with efficacy in animal models [72, 73]. Therefore, a phase II study in MSA patients is currently under consideration [74].

### Enhancing α-synuclein degradation

There is growing evidence that the autophagy-lysosomal pathway is affected in MSA [75]. One such pathway is the mammalian target of rapamycin complex (mTOR) pathway. Rapamycin, also known as sirolimus, is an immunosuppressant that specifically inhibits actions of mTOR by allosterically modulating access to the catalytic site of mTOR [76]. A recent proof-of-concept study demonstrated partial neuroprotection and reduction in α-synuclein aggregates in PLP-α-synuclein transgenic mice after treatment with rapamycin [77]. An additional preclinical study showed evidence of motor improvement, reduction of 4-hydroxynonenal-protein-adducts, and attenuation of synaptic injury in A53T α-synuclein transgenic mice [78]. However, isolated dysfunctional macroautophagy did not improve clearance of abnormal accumulation of α-synuclein *in vitro*. A phase II randomized controlled study in patients with MSA assessing efficacy of oral sirolimus on slowing disease progression was recently prematurely terminated because meeting the futility criteria (NCT03589976).

The antibiotic rifampicin inhibits the formation of α-synuclein fibrils and disaggregates fibrils already formed in MSA mouse models [80, 81]. A large phase III placebo controlled trial (NCT01287221) was terminated prematurely after a preplanned interim analysis of the primary endpoint (mean rate of change of UMSARS I score) revealed that futility criteria had been met [82].

Nilotinib is a compound acting as a tyrosine kinase Abelson (Abl) inhibitor approved for the treatment of chronic myeloid leukaemia. Preclinical evidence suggested that this drug can degrade misfolded α-synuclein by enhancing the autophagy-lysosomal pathway [83, 84] and reduce oxidative stress [85, 86]. Unfortunately, Nilotinib failed to show a disease modifying effect in mouse models of MSA [87]. However, findings in PD patients were observed controversial in clinical trials. A small open-label phase I clinical trial in patients with PD dementia and DLB showed positive safety and tolerability profiles [88] and phase II randomized-controlled trial confirmed reasonable drug safety and demonstrated effects on CSF levels of dopamine metabolites, α-synuclein oligomers, and tau in patients with PD [89]. However, another recent phase II trial demonstrated low CSF exposure and no efficacy after a 6-month treatment. These findings will guide trial development in patients with PD and MSA [90].

Lithium reduces α-synuclein aggregation and stimulates autophagy and neuroprotection in preclinical *in vivo* and *in vitro* models [91–93]. A phase II trial of lithium in patients with MSA was terminated due to severe adverse events discouraging further attempts of repurposing this drug in MSA [94].

### Preclinical developments

Molecular tweezers are nano-chaperones with open cavities able to bind with guest molecules with non-covalent binding or electrostatic effects [95]. Upon treatment with the molecular tweezer CLR01, a reduction of α-synuclein load and dose-dependent reduction in GCI density was reported in a transgenic mouse model of MSA [96, 97]. These encouraging findings suggest a potential for disease modification in MSA and other synucleinopathies; however, the low penetrance through the blood-brain-barrier is a concern that needs to be addressed in future preclinical studies.

NPT088, a fusion protein combining a human immunoglobulin backbone with a general amyloid interaction motif, is currently under active clinical development for Alzheimer’s disease (NCT03008161). The protein’s motif not only recognizes amyloid-beta and phosphorylated tau but also misfolded α-synuclein, markedly decreasing amounts of aggregated α-synuclein in a PD mouse model [98].

The small molecule SynuClean-D was identified by a high-throughput screening assay. First tests *in vitro* and in PD models showed inhibition of α-synuclein aggregation by binding to α-synuclein fibrils, disruption of amyloid fibrils, and prevention of dopaminergic neurons degeneration [99].

The c-Abl kinase inhibitor IkT-148009 is currently being studied in PD animal models. Plans are also being made to study it in MSA animal models [100]. A phase I clinical trial of IkT-148009 in healthy volunteers and patients with PD is currently underway (NCT04350177).

The neurosin Kallikrein-6 is a serine protease with the ability to cleave α-synuclein in the central nervous system (CNS). When transferred through a lentiviral vector, a reduction of α-synuclein accumulation was shown in DLB/PD transgenic mouse models [101]. In the study by Spencer et al., kallikrein-6 was modified by the R80Q mutation resulting in longer half-life and was fused with the protein apoB for an effective transport through the blood-brain barrier [102]. This neurosin (NR)-R80Q-apoB enrichment resulted in a reduction of α-synuclein accumulation in oligodendrocytes and astrocytes, and in an improvement of the myelin sheath formation in the corpus callosum of MBP-α-synuclein transgenic mice. Additionally, behavioral improvements in cognition and locomotor activity were shown. However, a recent study demonstrated that reduced activity of kallikrein-6 is more likely a compensatory response than the cause of α-synuclein accumulation in MSA [103].

### Targeting neuroinflammation

Widespread neuroinflammation and concomitant microglial activation are key histopathological findings in MSA paralleling neurodegeneration in brain areas affected by disease pathology [104]. Increasing evidence suggests that misfolded α-synuclein triggers microglial activation and astrogliosis in MSA and related α-synucleinopathies [4, 105–107]. Neuroimaging studies indicate severe neuroinflammation in MSA patients with a regional pattern consistent with the underlying MSA neuropathology [108]. Further, elevated levels of proinflammatory cytokines were previously reported [109]. However, it remains to be established whether neuroinflammation is a secondary consequence of neurodegeneration or an independent contributor to the pathophysiological cascade in MSA. Studies evaluating the disease-modifying potential of modulators of neuroinflammation were summarized in Table 2.

**Table 2:** Clinical trials targeting neuroinflammation

Target	Substance	Phase	Design	Primary outcome	Results	Comments
Inhibition of neuro-inflammation	**IVIG**	Phase II	OL	Number of AEs	Positive	Motor improvement (small sample size, short treatment period)
Inhibition of microglial activity	**Minocycline**	Phase II	RCT	UMSARS part II score	Negative	No motor improvement
Oxidative stress reduction	**Verdiperstat**	Phase III	RCT	Modified UMSARS total score	Negative	Failed in terms of primary and key secondary endpoints

IVIG, intravenous immunoglobulin; RCT, randomized-controlled trial; OL, open label trial; UMSARS, Unified Multiple System Atrophy rating scale.

### Clinical development

Minocycline, a tetracycline antibiotic, was shown to inhibit microglial activation and its downstream events such as secretion of pro-inflammatory cytokines [110, 111]. A phase II randomized controlled trial of minocycline administered to MSA patients for 48 weeks failed to demonstrate motor improvement or neuroprotective effects [111]. However, [^11^C](R)-PK11195 PET for *in vivo* evaluation of neuroinflammation demonstrated target engagement with a reduction of subcortical microglial activation in a subgroup of MSA patients [111].

Neuroinflammation and production of toxic cytokines in MSA provide evidence in favor of intravenous immunoglobulin (IVIG) therapy [112–114]. IVIG inhibit autoreactive T-cells, suppress autoantibodies and interfere with the production of cytokines [114]. Novak et al. studied the effects of IVIG infusions in an open-label pilot study in 9 MSA patients showing a decrease of UMSARS scores in majority of patients [114]. No changes on brain MRI and no serious adverse events were observed. Despite these positive signals, a larger, confirmatory study is required to establish the efficacy of IVIG therapy in MSA.

Myeloperoxidase (MPO) plays a key role in the production of reactive oxygen species by phagocytic cells [115–117]. Verdiperstat is a potent inhibitor of MPO suppressing microglial activation and improving motor function in a transgenic MSA mouse model [106]. However these effects failed to influence motor impairments in a mouse model of advanced MSA [118]. Several phase I studies evaluating verdiperstat in healthy subjects reported no safety concerns. A phase II study reported amelioration of microglial activation in patients with PD [119]. In MSA patients, a phase II study showed trends towards clinical efficacy (NCT02388295). A phase III randomized controlled trial has recently being finished and failed to meet its primary and key secondary endpoints including a modified UMSARS score, the Clinical Global Impression of Improvement (CGI-I) score and the MSA quality of life questionnaire (NCT03952806) [120].

### Preclinical development

The inflammatory protease caspase-1 promotes the aggregation of α-synuclein [121]. In a proof-of-concept study the caspase-1 inhibitor prodrug VX-765 ameliorated α-synuclein aggregation toxicity in transgenic MSA mice [122].

A combination of α-synuclein directed antibody and anti-inflammatory treatment was recently evaluated in a transgenic MSA mouse model [123]. CD5-D5 is a CNS penetrating single-chain antibody targeting α-synuclein. Lenalidomide, a small thalidomide derivative, marketed as an anticancer drug for multiple myeloma. The combined treatment showed a reduction of astrogliosis, microgliosis, as well as soluble and aggregated α-synuclein levels in transgenic MSA mice.

The immunomodulatory drug Fingolimod, currently marketed for treating multiple sclerosis, shows neuroprotective effects in different animal models by increasing brain-derived neurotrophic factors. The modified derivate FTY720-Mitoxy is known to increase expression of brain-derived neurotrophic factor (BDNF), glial-cell-line derived neurotrophic factor (GDNF), and nerve growth factor [124, 125]. Vidal-Martinez et al. reported a potent protective effect of FTY720-Mitoxy in CNP-α-synuclein transgenic MSA mice by reduction of motor disability and neuroinflammation, restoration of mitochondrial function and an increase of GDNF expression [126].

A synthetic microneurotrophin BNN-20 reduced microglial activation, increased BDNF and restored dopaminergic neurons even in advanced stages of neurodegeneration [127].

### Other selected neuroprotective strategies

Potential neuroprotective disease modifying therapies are summarized in Table 3.

**Table 3:** Clinical trials targeting Neuroprotection and neurotrophic support

Target	Substance	Phase	Design	Primary outcome	Results	Comments
FAF-1	KM-819	Phase I	RCT	Safety & tolerability	Safe & well tolerated	–
Lipidomic neurotoxicity	YTX-7739	Phase Ib	–	Safety & tolerability	–	Ongoing
IGF1 pathway	Intranasal insulin	Phase II	RCT	Verbal fluency total score	–	Motor improvement (only 1 MSA patient)
IGF1 pathway	Exendin-4	Phase II	OL	UMSARS part I & II score	–	Ongoing
Mitochondrial dysfunction	Coenzyme Q10	Phase II	RCT	UMSARS part II score	–	Ongoing
Neuronal/glial proliferation	Growth Hormone	Phase II	RCT	Safety & tolerability	Safe & well tolerated	Trend for less worsening of UMSARS [152]
Immuno-modulation, neuro-protection	MSCs	Phase II	RCT	UMSARS part II score	Positive	Only in MSA-C, imaging not done in all patients [156]
Mitochondrial dysfunction	Rasagiline	Phase II	RCT	UMSARS part I & II score	Negative	No motor improvement [160]
Neurotrophic support	Fluoxetine	Phase II	RCT	UMSARS part I & II score	Negative	No motor improvement [163]
Reduced excitotoxicity	Riluzole	Phase III	RCT	UPDRS part II & III	Negative	No motor improvement or survival rates [137]
NMDA-modulator	Tllsh2910	Phase III	RCT	SARA score	–	Ongoing

IVIG, intravenous immunoglobulin; RCT, randomized-controlled trial; OL, open label trial; UPDRS, Unified Parkinson’s Disease Rating Scale; SARA, Scale for the assessment and rating of ataxia; UMSARS, Unified Multiple System Atrophy Rating Scale; MSA, multiple system atrophy.

### Clinical development

#### KM-819

The protein FAS-associated factor 1 (FAF1) is expressed in cells to induce apoptosis. Studies have shown that these proteins are overexpressed in PD and therefore lead to increased neuronal cell death [128, 129]. KM-819 is an orally active small molecule drug developed as an inhibitor for FAF1. The first study in healthy volunteers showed promising results in safety and tolerability [130]. A phase II trial in PD and MSA patients is being considered [131].

#### YTX-7739

To screen for α-synuclein associated targets, a lipidomic analysis of its neurotoxicity was performed. This revealed that oleic acid, whose production is triggered by the stearoyl-CoA desaturase (SCD) has a neurotoxic effect on neurons [132, 133]. Therefore, the SCD inhibitor YTX-7739 is currently explored in a phase Ib proof-of-concept study [134].

#### Riluzole

Riluzole is a glutamate antagonist and the only approved DMT for amyotrophic lateral sclerosis [135]. By blocking sodium and potassium channels, the stimulation of glutamate receptors can be reduced and excitotoxicity induced neuronal death can be prevented. Preclinical studies in rat models of MSA showed a significant reduction of motor deficits and striatal lesion volume, suggesting a potential neuroprotective effect [136]. Seppi et al. carried out a randomized-controlled trial in a small group of 10 MSA patients revealing a lack of motor improvement [137]. A subsequent, large placebo-controlled trial in patients with MSA and PSP reported that riluzole had no effect on disease progression and survival [138].

#### Tllsh2910

N-methyl-D-aspartic acid (NMDA) receptors in the cerebellum play a role in motor learning and coordination [139]. Tllsh2910, a NMDA modulator, has been found to attenuate ataxic gait in a MSA mouse model. A phase III randomized-controlled single center study is currently recruiting patients with MSA-C (NCT03901638).

#### Insulin-like growth factor pathway

Insulin plays an important role in many neurodegenerative disorders due to its neuromodulatory, neurotrophic, and neuroprotective effects [140]. There is evidence that insulin-like growth factor-1 (IGF-1) signaling is impaired in PD and Alzheimer’s disease [141]. While a clinical study observed increased plasma insulin and IGF-1 concentrations in patients with MSA patients [142], reduced IGF-1 brain levels were observed in a transgenic mouse model of MSA [143, 144]. A pilot randomized placebo-controlled trial with intranasal insulin in 14 PD and 1 MSA patient showed an improvement of Hoehn & Yahr staging, UMSARS motor scores and verbal fluency without serious adverse events in treated patients [145]. Another promising antidiabetic drug being tested for MSA is the glucagon-like peptide agonist exendin-4 [144]. Bassil et al. evaluated exendin-4 treatment in transgenic MSA mice and observed increased insulin receptor density in the most severely affected brain regions, reduced monomeric α-synuclein load in the striatum and protective effect on survival of nigral dopamine neurons [144]. However, motor signs were not improved in transgenic mice. A phase II open label study on exendin-4 in patients with MSA is currently underway (NCT04431713).

#### Coenzyme Q10

Even though MSA is largely a sporadic disease, a causal relationship between COQ2 mutations and cerebellar-type MSA was established in Japanese patients [146]. These mutations lead to decreased production of Coenzyme Q10 (CoQ10), which is an electron carrier in the mitochondrial respiratory chain, and a potent antioxidant. *In vitro* studies with induced pluripotent stem cell-derived dopaminergic neurons from MSA patients reported reduced CoQ10 levels and up-regulation of several CoQ10 biosynthesis enzymes in MSA patients compared to healthy controls [147, 148]. These changes were partially rescued by CoQ10 supplementation [148]. A case report of high-dose ubiquinol treatment in a patient with COQ2 mutation and MSA-C reported no evidence of clinical or imaging benefit after 3 years treatment [149]. Nevertheless, a phase II randomized-controlled trial that already finished recruitment is currently ongoing in Japan (UMIN000031771).

#### Growth hormone

Growth hormone was shown to stimulate neuronal and glial proliferation and increase myelination and brain size [150]. In contrast, growth hormone deficiency is associated with impaired survival of new neurons and deficits in brain development and function [151, 152]. Along those lines, Holmberg et al. carried out a randomized-controlled trial with recombinant human growth hormone (r-hGH) in patients with MSA. After 12 months of subcutaneous treatment, no difference in the treatment effect between r-hGH-treated and placebo-treated MSA patients was observed, although the small sample size was a significant limitation of this study [152].

#### Mesenchymal stem cells

Because of their immunomodulatory and neuroprotective effects, mesenchymal stem cells (MSCs) have been the focus of a potential MSA therapy for over a decade now. The first clinical trial was an open-label monocentric study assessing feasibility and safety of intra-arterial MSCs therapy showing promising results [153, 154]. In 2011, Stemberger et al. confirmed potential neuroprotective effects of MSCs in a transgenic mouse model of MSA [155]. A phase II randomized placebo-controlled study reported attenuated UMSARS part II score progression in MSA-C patients receiving autologous bone marrow derived MSCs via intra-arterial or intravenous routes compared to patients receiving placebo [156]. However, procedural related adverse events (small ischemic brain lesions upon intra-arterial infusion) raised safety concerns. This has prompted the conduct of another phase I trial revisiting the safety and tolerability of intra-arterial (carotid arteries) injection of autologous bone marrow-derived mesenchymal stem cells in MSA-C patients which was recently completed in South Korea; the results have not been published so far (NCT03265444).

Singer et al. in 2019 demonstrated slowing of motor progression compared to a historical cohort through intrathecal injections of autologous fat tissue derived MSCs [157]. The adverse event rate increased with higher doses with patients developing low back or posterior leg pain, associated with thickening/MRI enhancement of lumbar nerve roots. Otherwise, this therapy proved to be safe and well tolerated encouraging further clinical development.

#### Rasagiline

The irreversible monoamine oxidase-B (MAO-B) inhibitor rasagiline shows symptomatic benefits and a possible disease-modifying effect in PD patients by modulation of the mitochondrial metabolism [158]. Preclinical studies in a transgenic mouse model of MSA revealed motor improvement, reduction of GCI load and neuronal protection [159]. However, a multicenter phase II randomized placebo-controlled clinical trial of rasagiline 1 mg/day in MSA-P patients did not show clinical benefits [160].

#### Selective serotonin-reuptake inhibitors

The neurotrophic factors GDNF and BDNF play an important role in neuroprotection. Selective serotonin-reuptake inhibitors (SSRI), currently used as anti-depressants, are reported to have a positive impact on neurotrophic factor expression. In a transgenic MSA mouse model fluoxetine has been shown to increase GDNF and BDNF levels and to suppress pro-inflammatory cytokines [161, 162]. A phase II randomized placebo-controlled trial of fluoxetine in MSA patients failed to demonstrate fluoxetine superiority over placebo on the total UMSARS score, whereas trends in motor and emotional secondary/exploratory outcomes deserve further investigation [163]. However, a retrospective long-term analysis of over 600 patients with MSA revealed that patients treated with any SSRI did not differ from patients never treated with SSRI in survival, but manifested parkinsonism and falls more frequently [164].

Another study around GDNF is currently recruiting in order to evaluate the safety and potential clinical effects of bilateral imaging-guided infusion of AAV2-GDNF into putamen of patients with MSA in a randomized placebo-controlled phase I trial (NCT04680065).

### Preclinical development

#### Benztropine

The formation and repair of myelin is the main task of oligodendrocytes [165]. Alpha-synuclein accumulation in oligodendrocytes leads to demyelination resulting in axonal dysfunction and neuronal loss. Ettle et al. used the pro-myelinating activity of the muscarinic acetylcholine receptor antagonist benztropine attempting to reverse myelination deficits in different preclinical models of MSA. This experiment showed restoration of the α-synuclein-induced myelination of stem cell-derived oligodendrocytes and prevented neuronal loss in transgenic MSA mice [166]. Although benztropine has been used for decades in clinical neurology, it is debatable whether demyelination is a major target in MSA.

#### Monophosphoryl lipid A

Toll-like receptors (TLR) play an important role for innate immune response. Recently, TLR4 was identified as an important mediator of endogenous α-synuclein clearance by microglia [167]. Deficits of functional TLR4 resulted in increased α-synuclein accumulation, aggravation of the motor disability, and nigral degeneration in double MSA transgenic mice [168]. Preclinical studies in transgenic MSA mice treated with the TLR4 agonist monophosphoryl lipid A revealed an increased microglial α-synuclein uptake, significant motor improvement, rescue of nigral dopaminergic and striatal neurons, and region-specific reduction of the density of GCI in the absence of a marked systemic inflammatory response [169]. This approach offers an interesting option to fortify the endogenous mechanisms of α-synuclein clearance.

#### Sodium phenylbutyrate

Targeting MSA by its epigenetic roots, Sturm et al. described the hypothesis of a possible interference of α-synuclein with histone acetylation in glial and neuronal cells causing inhibition of acetylation and resulting in neurotoxicity [170, 171]. The pan-histone deacetylase inhibitor sodium phenylbutyrate showed a significant improvement of motor behavior and survival of nigral neurons in PLP–α-synuclein mice [171].

### Trial design and outcome measures

Although large efforts were undertaken to conduct disease-modifying trials in MSA, improvements in trial methodology are still required. As highlighted throughout the entire review, we can only speculate on the reasons of failure of previous clinical trials in MSA. It is likely that the trials failed because of lacking efficacy of the studied compound; however, other issues associated with the trial design including too short follow-up periods, insufficient sample sizes, and high drop-out rates may have also had an impact on the outcome of the studies. Therefore, it is important to perform careful sample size estimates in the study planning and introduce measures to improve patient retention and study adherence. Additionally, the novel International Parkinson and Movement Disorder Society (MDS) criteria for the diagnosis of MSA will improve diagnostic accuracy in established as well as early stage disease enabling researchers to recruit more homogenous patient cohorts and earlier stage MSA patients.

Furthermore, there is still an unmet need of reliable surrogate biomarkers defining disease progression in MSA beyond clinical scales. Although much effort is taken here, we need to better define the natural history of this disease and do more research on possible surrogate biomarkers of disease progression including multimodal MRI and biofluid markers. Despite contradictory results in previous studies [172], CSF α-synuclein as well as markers of neurodegeneration (e.g., neurofilament) and glial dysfunction (e.g., GFAP) may be helpful. Recently, a novel PET-tracer has been proved to distinguish MSA from other synucleinopathies demonstrating promising potential as an imaging outcome parameter for future studies [173]. Finally, a clinical outcome assessment instrument capturing patient-cantered milestones will be essential and, recently, efforts attempting to improve and address shortcomings of the current version of the UMSARS were intensified and an expert task force working on a revised UMSARS was established within the MDS.

## CONCLUSION AND OUTLOOK

The therapeutic management of patients with MSA remains largely frustrating with a lack of disease-modifying agents and symptomatic therapies that only offer transient and partial benefit to a subgroup of patients. Hence, there is an urgent unmet need for disease modifying therapies in MSA. Over the past two decades preclinical MSA models were developed to thoroughly characterize molecular changes and underlying neuropathophysiological events in MSA. These previous research efforts included neurotoxin-based animal models [174], targeted overexpression of α-synuclein through transgenic modifications [175] or viral vectors [176]. More recent studies performed intracerebral inoculation of MSA brain extracts into transgenic mice in an attempt to study seeding properties of MSA-specific α-synuclein strains. Despite the incomplete recapitulation of human MSA pathology [175, 177] preclinical studies have taught us important lessons on the pathophysiological cascade of MSA and secondary changes caused by aberrant α-synuclein aggregation [178]. These models opened an avenue to develop novel agents with disease-modifying effects and, preclinically, for confirmation of target engagement. However, as highlighted above, several candidate therapies showed preclinical evidence of neuroprotection, but they did not translate into a clinical benefit in large-scale interventional trials raising questions on to what extent preclinical target engagement can predict a clinical benefit.

Although previous therapies targeting α-synuclein failed to slow disease progression [82, 179], there is converging and convincing preclinical evidence that α-synuclein is a key contributor to disease spread and that its toxic effects promote cell death in MSA [8, 22]. Hence, reduction of CNS α-synuclein load exploiting highly effective novel treatment strategies including immunotherapies, genetic modification and enhancing α-synuclein clearance (through disaggregation or enhancing the autophagy-lysosomal pathway) remains a promising approach to disease modification.

Neuroinflammation and its secondary consequences including oxidative stress are other key contributors to neurodegeneration in MSA. Despite evidence of target engagement on PET imaging, minocycline (an antibiotic with anti-inflammatory effects) failed to improve motor function in a small-scale study [111]. More recently, highly specific drugs tackling key enzymes were studied preclinically. Among these, an irreversible inhibitor of myeloperoxidase evolved to the clinical development stage. Based on encouraging results from early phase clinical studies on MPO inhibitors, a large-scale, phase III study has recently been completed but unfortunately, the trial failed at primary and key secondary endpoints.

Stimulation of neuronal and glial proliferation, enhancing myelination through trophic support and cell replacement therapies are areas that are currently being pursued. An early study with recombinant growth hormone was negative; however, there was a numerical trend towards motor improvement in the growth hormone treated group. The small sample size of the study impedes a final conclusion. Although concerns of potential adverse events were raised, mesenchymal stem cells mediated neuroprotective effects in a small-scale study in MSA-C patients and larger, confirmatory studies are currently underway.

In summary, the large number of ongoing preclinical and clinical efforts with promising interventions foster hope for the discovery of a disease-modifying agent.
